# Quality of Life Changes Among Breast Cancer Survivors Following Mastectomy

**DOI:** 10.7759/cureus.97973

**Published:** 2025-11-27

**Authors:** Elaine Chi, Rishank Chillakuru, Latha Ganti

**Affiliations:** 1 Biology, Trinity Preparatory School, Winter Park, USA; 2 Biomedical Engineering, Case Western Reserve University, Cleveland, USA; 3 Medical Science, The Warren Alpert Medical School of Brown University, Providence, USA

**Keywords:** breast cancer, quality of life, quality of life in breast cancer patient, survey analysis, web-based survey

## Abstract

Background

Breast cancer is one of the most common cancers among women in the United States, and can lead to negative impacts on patient self-esteem. This study aims to provide a deeper understanding of the struggles of breast cancer survivors post-mastectomy in all aspects of their daily lives to encourage the provision of effective support.

Methods

Data was collected through a 20-question, nationwide, anonymous web-based survey. Questions assessed challenges in life after mastectomy and individual respondents' perceptions.

Results

A total of 100 responses were collected from breast cancer survivors who underwent mastectomy. Results indicate positive changes in patient health following recovery, with many survivors noting the development of new healthy habits. However, most participants also reported feeling less physically capable after treatment, resulting in a decreased ability to pursue hobbies and perform daily tasks. These physical limitations are linked to difficulty in survivors’ abilities to advance in their careers, which may also be connected to increased financial burden during and after treatment.

Conclusion

In this nationwide survey of breast cancer survivors who underwent mastectomy, many survey participants indicated feelings of isolation, which, along with lowered self-esteem post-surgery, contributes to decreased mental health. Reconstructive surgery also plays a large role in survivors’ quality of life, with participants who underwent breast reconstruction indicating greater satisfaction with their current lifestyles.

## Introduction

First documented 3000-5000 years ago, breast cancer has remained a pressing global health concern in spite of medical advancements, with global incidence rates rising at a rate of more than 1% per year from 2011 to 2020 [1-3]. In the United States, approximately one in eight women will be diagnosed with breast cancer during their lifetime, making it the second most commonly diagnosed cancer, following skin cancer in women [4]. Globally, however, breast cancer is the most frequently diagnosed cancer in women across all populations [5].

Today, there are many different therapies and treatments for breast cancer, including chemotherapy, radiotherapy, endocrine therapy, immunotherapy, and surgery [6]. The treatments used depend on many factors, including age, genetics, patient preference, and cancer type, and it should be noted that different treatments are often used in conjunction with one another for optimal results [7-9].

Mastectomy is a form of surgical treatment of breast cancer in which the breast is either partially or entirely removed. There are several different types of mastectomy, including radical mastectomy, modified radical mastectomy, total mastectomy (also referred to as simple mastectomy), skin-sparing mastectomy, and nipple-sparing mastectomy [10].

Studies show that 30% to 40% of breast cancer patients in the United States undergo mastectomy as part of their treatment plan [11]. There are several reasons why breast cancer patients may choose to undergo mastectomy, the most common being prevention of cancer recurrence [12]. However, mastectomies often also present with postoperative side effects, including limited shoulder and upper-limb mobility and chronic pain [13,14].

Patients who have undergone mastectomy often undergo reconstructive surgery, a procedure in which the shape of the breast is rebuilt after mastectomy. Breast reconstruction can be done immediately after mastectomy, during the same surgery, or at a later date [15]. There are many benefits to undergoing breast reconstruction surgery, such as increased physical well-being and comfort, though the largest motivator for reconstructive surgery following mastectomy is improved body image [16,17].

Studies have concluded that mastectomies often have lasting, detrimental effects on patients’ mental well-being, causing drops in self-confidence and social isolation [18]. This study aims to provide a deeper understanding of the struggles of breast cancer survivors post-mastectomy, with the goal of encouraging action towards assisting survivors in remedying these struggles and providing the necessary information for effective support.

## Materials and methods

Inclusion and exclusion criteria

Inclusion criteria for respondents were adults aged 18 years or older who were residents of the United States who underwent a mastectomy procedure for breast cancer and gave consent to participate in the survey. Exclusion criteria were not meeting the inclusion criteria, and incomplete survey responses or duplicate entries.

Survey instrument

The survey consisted of 20 questions designed to assess various aspects of the respondents’ lives after mastectomy, including mental well-being and lifestyle choices. The questions were a mix of multiple-choice, Likert scale, and open-ended formats. The survey was pilot tested on a sample of 50 individuals to ensure clarity and reliability.

Data collection

Data were collected anonymously, and no personally identifiable information was gathered. Participants were informed about the purpose of the study and provided informed consent electronically before starting the survey. The average time to complete the survey was approximately 15 minutes.

Data analysis

**Figure 1:**
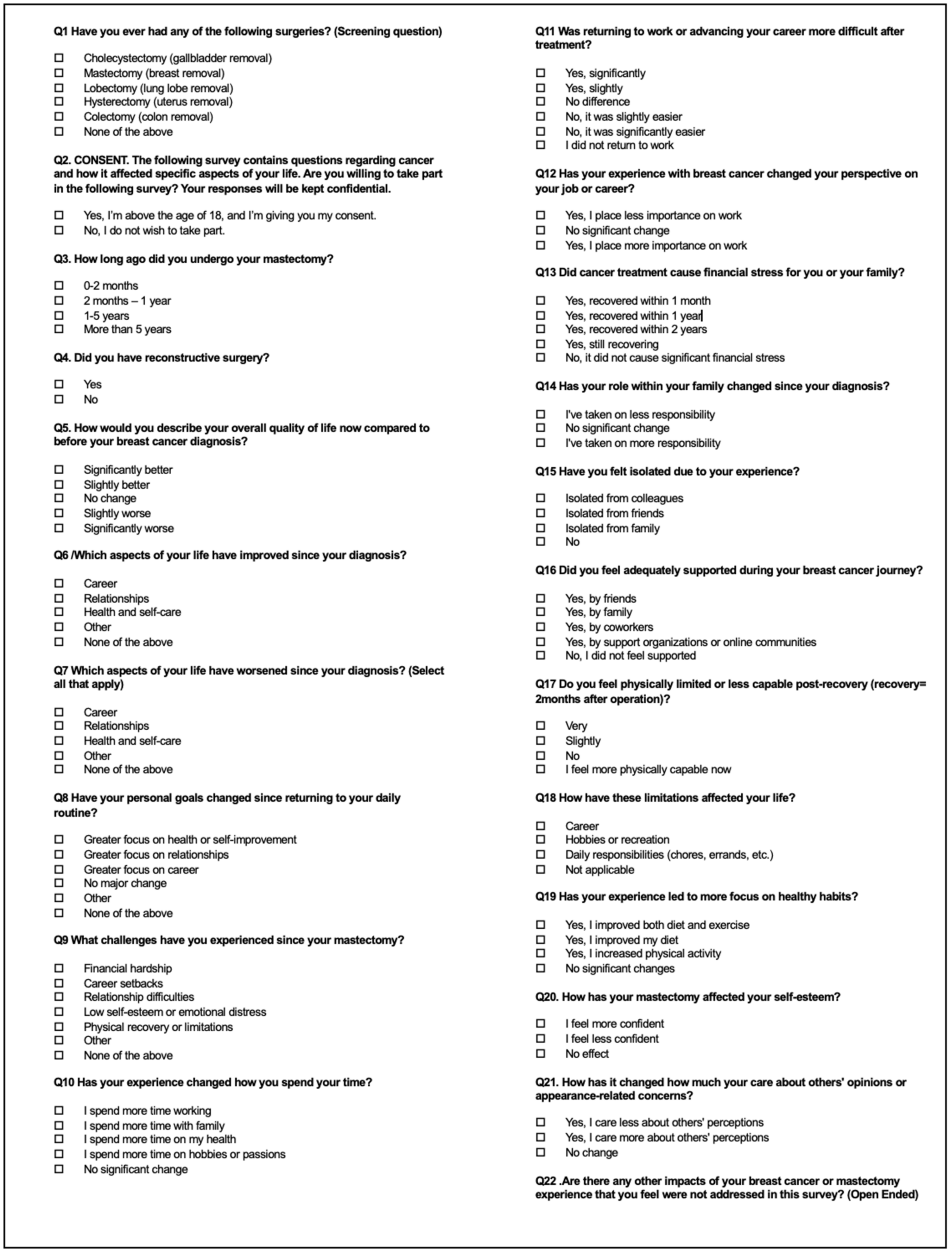
Survey instrument

A total of 100 de-identified survey responses were obtained from post-mastectomy breast cancer survivors regarding their experiences post-recovery. Survey responses were sorted and visualized using Microsoft Excel (Microsoft Corp., Redmond, WA, USA) and Power BI (Microsoft Corp.). A copy of the survey instrument is depicted in Figure 1.

Ethical considerations

As survey results were obtained in a de-identified manner without the ability of the study authors to ever identify the individuals, the study fulfills the National Institutes of Health criteria for non-human subject research, and was issued a non-human subjects determination by Brown University. The study adhered to the ethical guidelines outlined in the Declaration of Helsinki.

## Results

In the cohort of 100 breast cancer survivors, 48% were male, and 52% were female. The median age of all participants was 42, with a maximum age of 85 and a minimum age of 22 years. The interquartile range was 25 years, ranging from 34.5 to 59.5. Race distribution indicated 56% of participants were White individuals, 16% of participants were Hispanic individuals, 13% of participants were Asian individuals, and 12% of participants were Black individuals. Three percent chose not to provide their race data.

**Figure 2:**
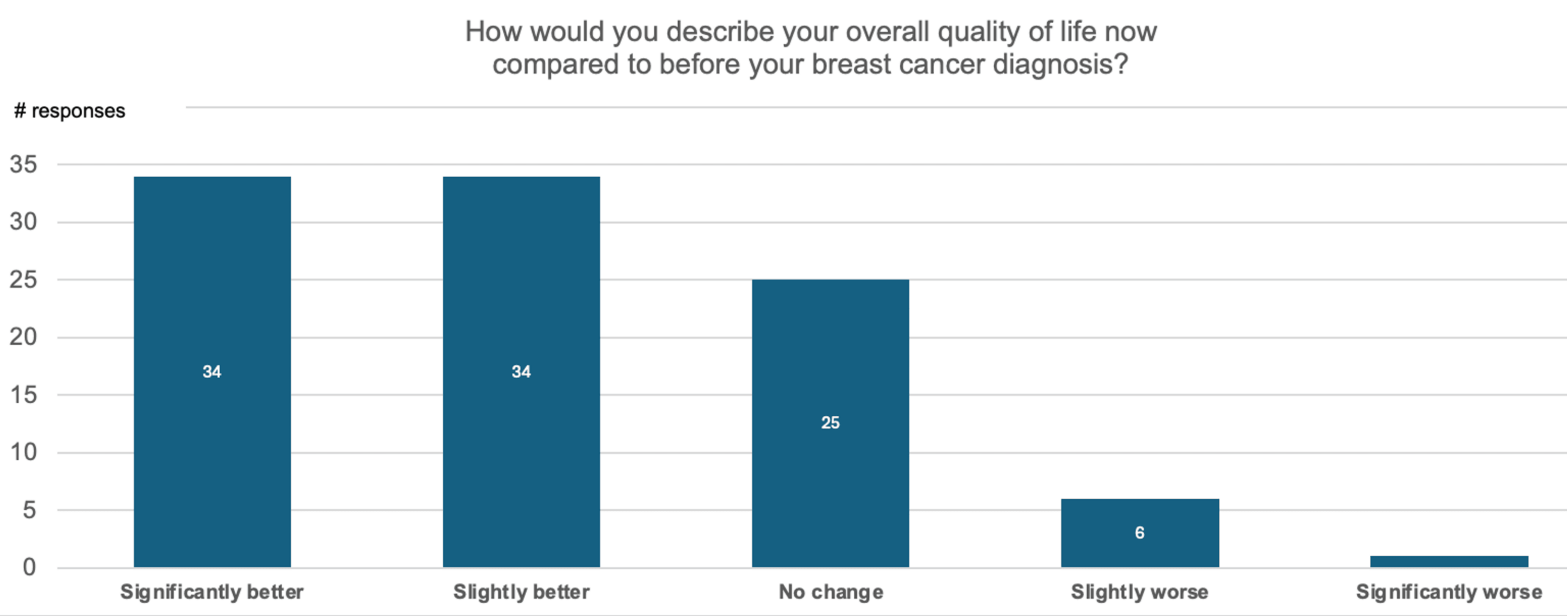
Changes in overall quality of life (QoL)

Figure 2 is a visualization of patient responses to the question “How would you describe your overall quality of life (QoL) now compared to before diagnosis?”

Sixty-eight percent of participants expressed that they believe their current QoL, after breast cancer treatment, is better than before their diagnosis. Only 7% believe their QoL to be worse, with the remaining 25% of participants expressing that their QoL did not experience any notable change.

When asked, “Which aspects of your life have improved since your diagnosis? (Select all that apply),” 72% of participants expressed that the health and self-care aspects of their lives improved, 54% expressed that their relationships improved, and 36% expressed that their careers improved. A total of 36% chose the option “none of the above,” indicating that neither their career, relationship, nor health has improved since their diagnosis. When asked, “Which aspects of your life have worsened since your diagnosis? (select all that apply),” 26% of participants identified their careers, 42% identified their relationships, and 58% identified their health and self-care. It should be noted that several participants selected “health and self-care” as an aspect that both improved and worsened.

**Figure 3:**
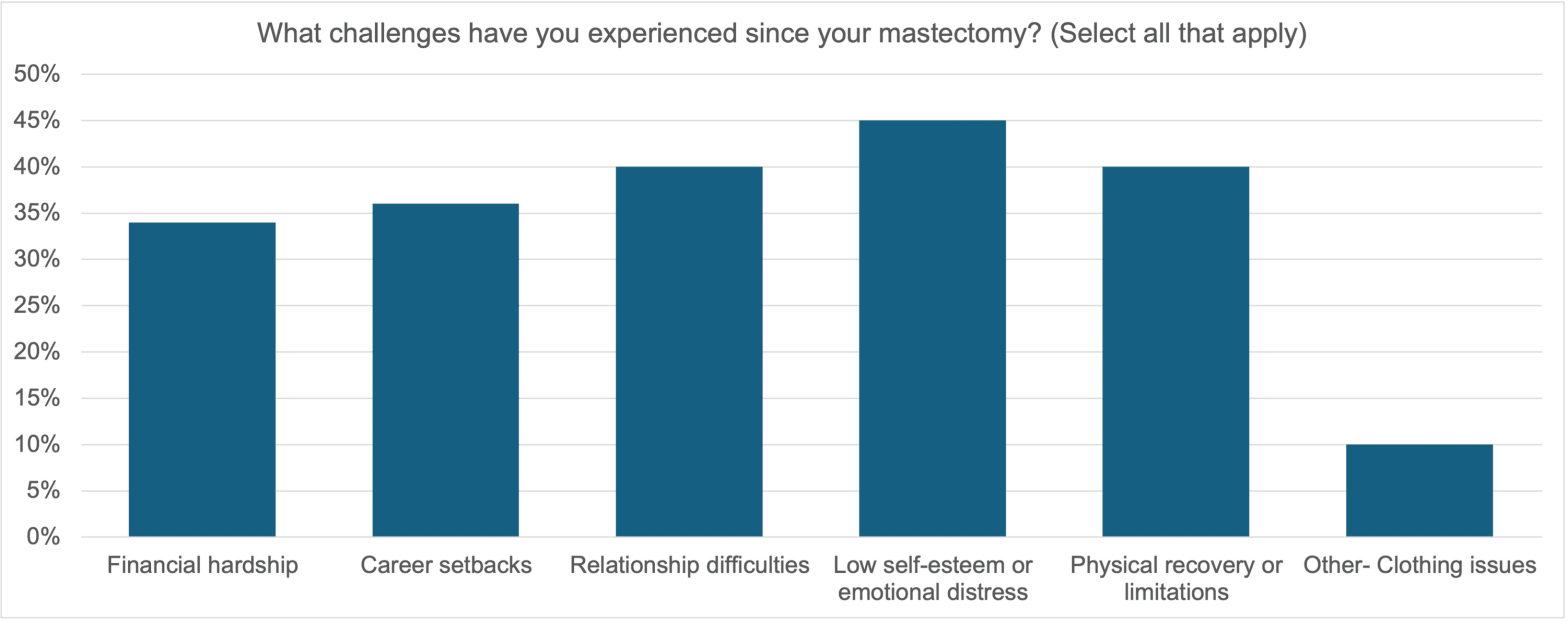
Challenges experienced following mastectomy

Figure 3 shows participant responses to the question “What challenges have you experienced since your mastectomy?"

Thirty-four percent of participants indicated they had financial struggles, 36% indicated that they had career setbacks, 40% indicated they had relationship difficulties, 45% indicated that their self-esteem decreased, and 40% indicated that they struggled to recover physically.

**Figure 4:**
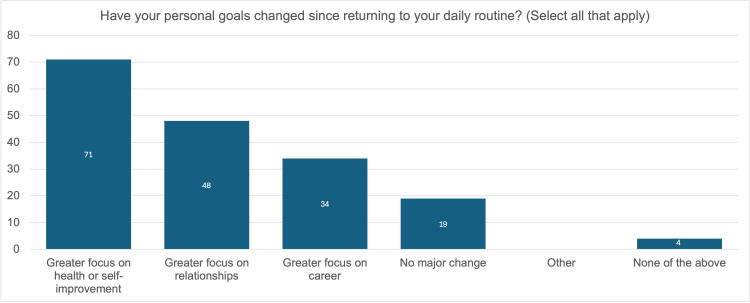
Changes in goals following recovery

Figure 4 is a visualization of responses to the question, “Have your personal goals changed since returning to your daily routine? (select all that apply).” An overwhelming majority of participants experienced a change in goals, with only 19% of participants indicating their goals remained the same. Seventy-one percent of participants indicated they now place more emphasis on health and self-improvement. Forty-eight percent of participants indicated that their goals now focus more on their relationships. Thirty-four percent of participants indicated that their goals are now more career-focused.

**Figure 5:**
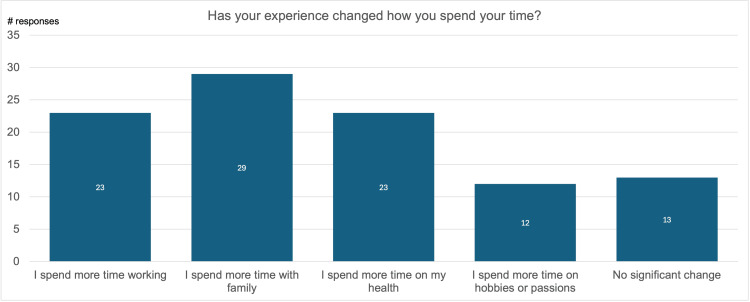
How respondents spend their time after mastectomy

Next, participants were asked, “Has your experience changed how you spend your time?” In response, 23% of participants reported that they now spend more time working, 29% reported that they now spend more time with their families, 23% reported that they now spend more time taking care of their health, and 12% reported that they now spend more time on their hobbies. Only 13% indicated no significant shift in their allocation of time (Figure 5).

**Figure 6:**
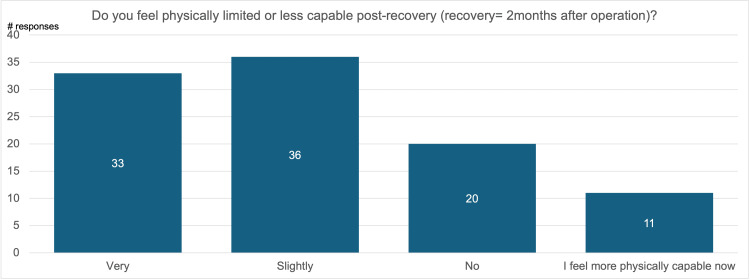
Physical limitations after recovery

Figure 6 is a visualization of patient responses to the question “Do you feel physically limited or less capable post-recovery?” Sixty-nine percent of participants felt less physically capable after treatment compared to before their diagnosis, with 33% indicating they felt very limited and 26% indicating they felt slightly limited. Twenty percent of participants expressed that they did not notice any significant change in their physical capabilities, and 11% expressed that they feel more capable than before their diagnosis.

As a follow-up question, participants were then asked, “How have these limitations affected your life? (select all that apply).” In response, 25% of participants who indicated that they noticed new physical limitations expressed that it affected their career, 19% expressed that it affected their participation in hobbies or recreational activities, and 33% expressed that it affected their ability to complete their daily responsibilities, such as household chores and errands.

**Figure 7:**
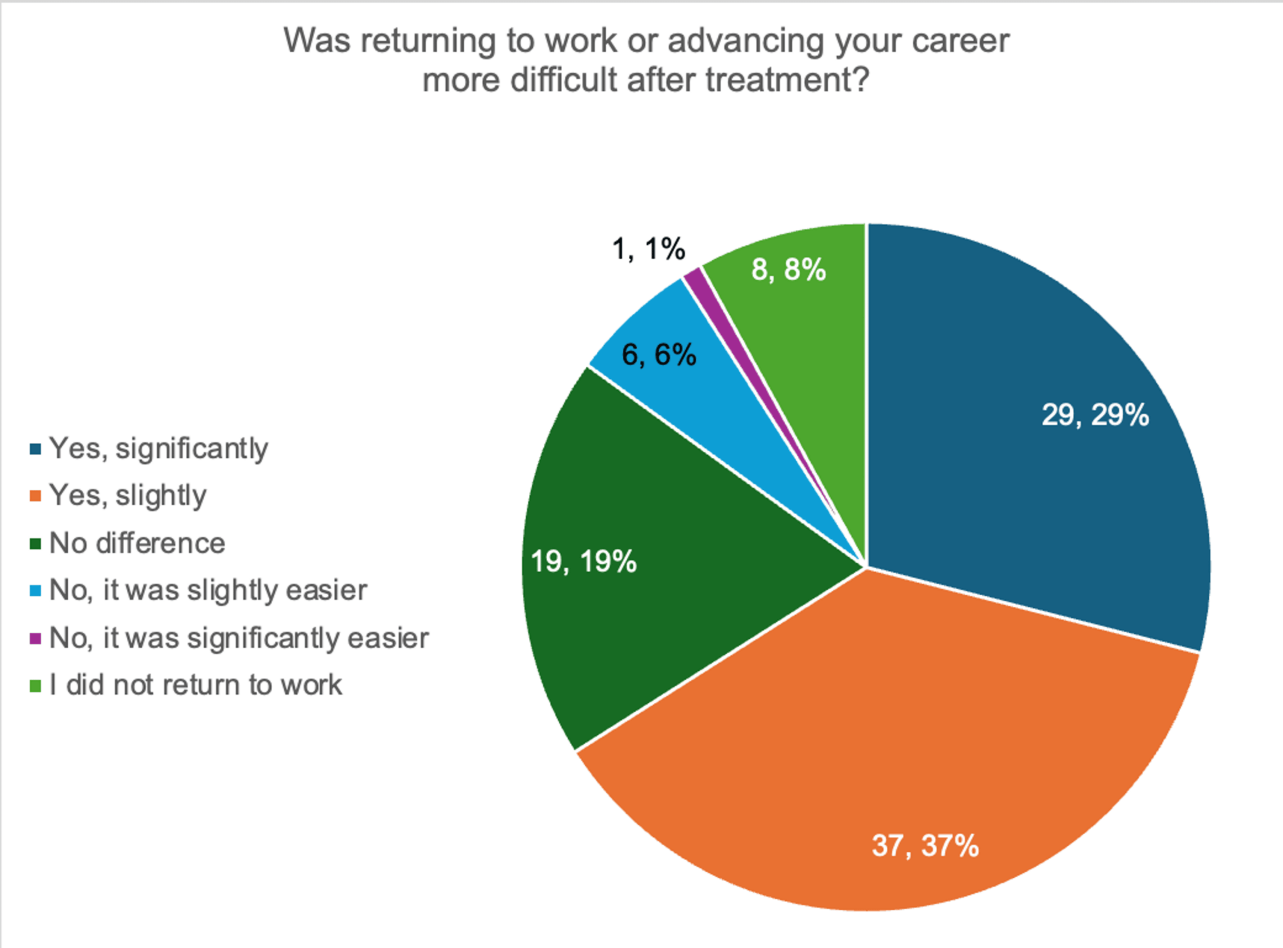
Difficulty in career experienced after treatment

Figure 7 displays participant responses to the question, “Was returning to work or advancing your career more difficult after treatment?” Sixty-six percent of participants indicated that they found it harder to make progress in their careers after returning to work. Twenty-nine percent found it significantly more difficult compared to before their diagnosis, while 37% found it slightly more difficult. Eight percent of participants did not return to work following treatment. Twenty-nine percent indicated that they felt no difference, and only 7% indicated that advancing in their career was easier after their experience with breast cancer compared to before their diagnosis.

**Figure 8:**
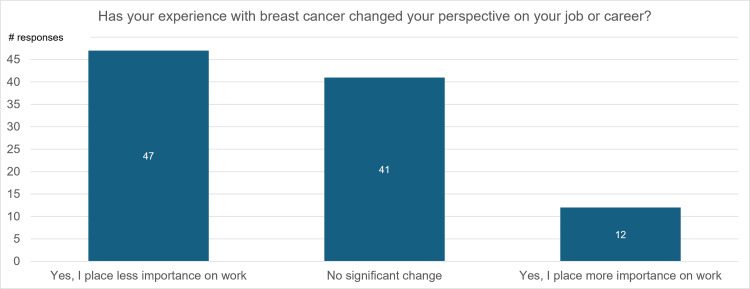
Responses on whether their breast cancer journey changed respondents' perspectives on their job or career

Figure 8 visualizes responses to the question “Has your experience with breast cancer changed your perspective on your job or career?” Forty-seven percent of participants reported that they now place less importance on work. Forty-one percent of participants reported that there was no significant change in how much they prioritized their careers. Only 12% indicated that they now place more importance on working.

**Figure 9:**
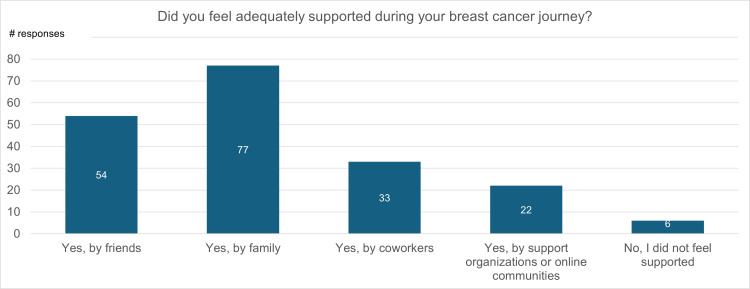
Adequacy of support during the breast cancer journey

Figure 9 displays participant responses when asked, “Did you feel adequately supported during your breast cancer journey? (select all that apply).” Fifty-four percent of participants reported that they felt adequately supported by friends, 70% felt supported by family, 33% felt supported by their coworkers, and 22% felt supported by breast cancer organizations or online communities. Only 6% did not feel adequately supported during their journey.

**Figure 10:**
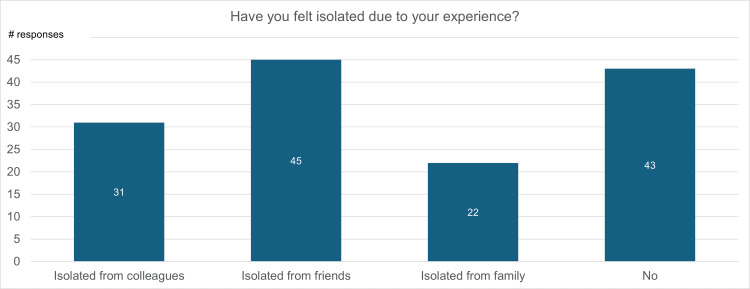
Isolation due to breast cancer experience

Figure 10 is a visualization of participant responses to the question “Have you felt isolated due to your (breast cancer) experience? (select all that apply).” Thirty-one percent of participants felt isolated from their colleagues, 45% felt isolated from their friends, and 22% felt isolated from their families due to their experience with breast cancer. Forty-three percent of participants did not feel isolated from any of the mentioned groups.

**Figure 11:**
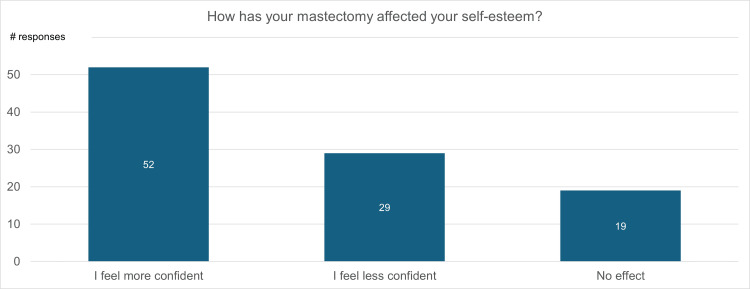
Effect of mastectomy on self-esteem categorized by sex

Figure 11 is a visualization of participant responses when asked, “How has your mastectomy affected your self-esteem?” sorted by gender. In total, 52% of participants felt more confident following mastectomy, 29% felt less confident, and 19% expressed that their experience did not affect their self-esteem or confidence. When broken down by gender, 29% of female participants felt more confident after a mastectomy, 38% of female participants felt less confident, and 33% of female participants’ self-esteem was not affected. For men, 77% felt more confident, 19% felt less confident, and 4% felt no effect on their self-confidence.

**Figure 12:**
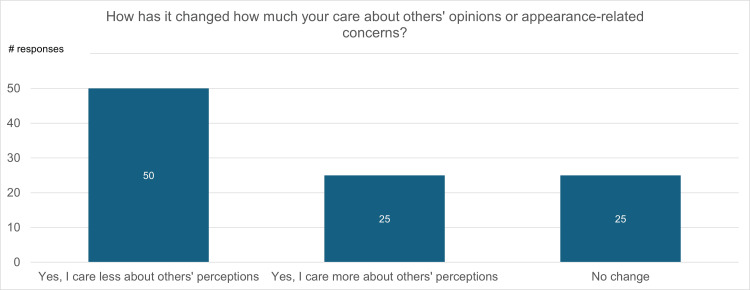
Impact of mastectomy on concerns about appearance or judgment

Figure 12 is a visualization of participant responses to the question “How has your experience changed how much you care about others' opinions about you?” In response, 50% of participants indicated that they now care less about others’ perceptions, 25% indicated that they care more about others’ perceptions, and the remaining 25% indicated that there was no change in how much they care about others’ perceptions of them.

**Figure 13:**
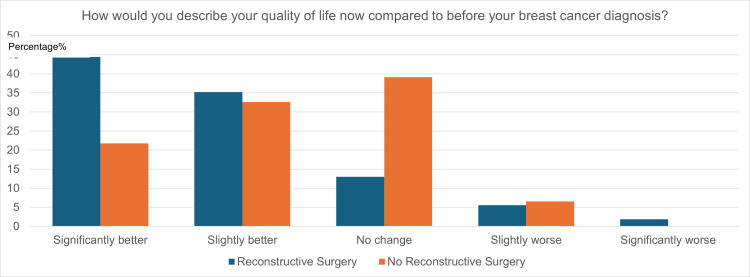
Quality of life (QoL) in respondents with versus without reconstructive surgery

Of the entire cohort of 100 breast cancer survivors, 54% underwent breast reconstruction surgery in addition to mastectomy. Forty-six percent did not undergo reconstructive surgery. Figure 13 shows participants’ responses when asked, “How would you describe your QoL now compared to before your breast cancer diagnosis?”

Of participants who underwent reconstructive surgery, 80% believe their current QoL is better than before their breast cancer diagnosis, with 45% believing their QoL to be significantly better and 35% believing it to be slightly better. Thirteen percent of participants believe their QoL did not change, 6% believe their QoL to be slightly worse, and 2% believe their QoL to be significantly worse. Of participants who did not undergo reconstructive surgery, 22% reported their QoL to be significantly better, 33% reported it to be slightly better, 39% reported it to be the same, and 6% reported it to be slightly worse.

**Figure 14:**
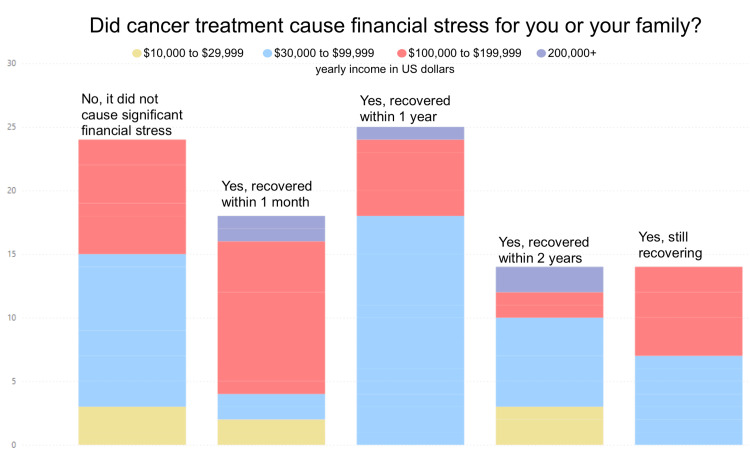
Financial impact of treatment categorized by income

Figure 14 shows participant responses to the question “Did cancer treatment cause financial stress for you or your family?” Results were categorized by income. No correlation was found between financial stress and household income.

## Discussion

Breast cancer and treatment cause many changes in patients’ lives. To start, a majority of survey participants indicated that they believe their overall QoL now is better than before their diagnosis, and the aspect that the most participants considered positively was “health and self-care”. Greater than 70% of participants thought health or self-care improved, despite experiencing breast cancer and the side effects of its treatment. This can be attributed to patients’ change in mindset regarding their bodies following a serious health complication, where they are now more likely to view their health and physical well-being as a priority, having experienced severe illness. Consequently, breast cancer survivors are likely to develop healthy habits and make efforts to stay physically fit. Sixty percent of participants stated that they improved both their diet and exercise, and another 25% indicated that they improved at least one of the two. Another notable aspect of many breast cancer survivors’ efforts to improve or maintain a healthy body is the fear of cancer recurrence. Though mastectomy is a procedure meant to minimize the risk of cancer recurrence, survey participants still indicated that they are worried about getting cancer again [19]. Physical activity has been shown to be positively associated with decreased risk of cancer recurrence, making prevention a strong motivator for physical activity and healthy habits [20]. This is also a potential factor in breast cancer survivors’ mental health, as studies have shown a significant correlation between fear of cancer recurrence and mental health issues such as depression and anxiety [21].

Although a majority of participants indicated positive changes in their health overall, most participants, including those who believed their health improved, still felt the physical effects of breast cancer and treatment. Many participants felt less physically capable compared to before their diagnosis, with male participants reporting this experience more frequently and to a higher severity. The physician's limitations expressed could be due to pain syndromes, joint stiffness, limited joint mobility, or swelling, all of which are potential side effects following mastectomy [22]. The decrease in physical capability noted by survey participants can also be explained by cancer-related fatigue, a symptom not uncommon in cancer patients that often persists after treatment [23]. Survey responses further indicate that these limitations had consequences on patients’ daily lives, affecting their careers, ability to do daily chores and errands, and ability to pursue hobbies.

Participant responses to the survey showed strong indications of career-related changes in post-mastectomy breast cancer survivors. When asked about their views on their careers, 47% of participants reported that they now perceive their career as less important than before their diagnosis (Figure 7). This is consistent with the shifting of personal goals after recovery towards health and relationships, as displayed in Figure 3. However, the decrease in career involvement cannot be attributed solely to a change in mindset and personal goals. A majority of survey participants expressed that they found progressing in their careers more difficult after returning to work (Figure 6), a pattern that was more significant in male participants than female participants. This could be associated with physical limitations experienced by survivors, as mentioned above. Changes in breast cancer survivors’ careers are especially significant due to the disease’s prevalence in the young, working population. Past studies have found the median age of diagnosis in breast cancer patients to be around 60 years of age (though the number can be higher or lower depending on factors such as race), meaning more than half of breast cancer patients are still of working age [24]. Unemployment or reduced career involvement following cancer treatment can decrease income in a time when patients and families already exhibit increased spending for treatment, heightening the financial pressure on breast cancer survivors and their families. This financial pressure is associated with anxiety, emotional distress, and decreased QoL [25].

Breast cancer treatment cost, though varied depending on factors such as cancer stage and treatment options, is a significant financial burden for many breast cancer survivors and their families [26]. Only 28% of survey participants expressed that breast cancer treatment was not a significant financial burden for them or their families. The remaining 72% expressed that cancer treatment did cause financial stress for them or their family. Upon further analysis by income range, there was no apparent association between income group and perceived amount of time it took to recover financially from cancer treatment (Figure 13). This is likely due to the presence of numerous external factors in the financial burden of treatment outside of yearly income. Treatment length and cost, insurance coverage, financial aid, savings, and living costs all affect financial stress.

Breast cancer survivors also often notice changes in their relationships throughout and after treatment. Ninety-four percent of survey participants expressed that they felt supported during their breast cancer journey by either their friends, family, or colleagues. However, 57% of participants felt isolated from at least one of these groups during or following their experience with breast cancer (Figure 9). This indicates that, regardless of support received, breast cancer survivors may experience negative social and psychological consequences that can lead to decreased QoL.

Breast cancer and mastectomy have extensive impacts on patients’ mental health. Of the 100 survey participants, 45% expressed that a significant challenge they faced after mastectomy was low self-esteem (Figure 2). Upon further analysis, the impact of mastectomy on patients’ self-esteem is strongly linked to gender. The percentage of female participants who lost confidence after mastectomy was double the percentage of male participants (Figure 10). This could be due to the breast’s perceived ties to femininity, beauty, and motherhood, as well as the pressure of societal expectations and norms. Furthermore, changes in self-esteem are also tied to patient age. Studies indicate that women over the age of 50 are less likely to develop body image insecurities following mastectomy [27].

Patient QoL was also analyzed based on whether reconstructive surgery was performed. From Figure 12, reconstructive surgery plays a large role in patients’ QoL post-mastectomy, as those who underwent breast reconstruction were noticeably more likely to describe their current QoL positively. This is consistent with past studies, which have concluded that reconstructive surgery has positive benefits both cosmetically and psychologically [28]. Past studies have achieved similar results, with breast reconstruction patients expressing greater satisfaction with their body image post-surgery and less impact on their self-esteem and sexual life [29].

Finally, survey participants were given the opportunity to share significant details about their experiences post-mastectomy that were not addressed by the survey. Most notably, several participants expressed their frustration regarding the availability of clothing designed for those who had undergone a mastectomy, with one person stating, “Clothes don't fit right, [and] it doesn't help self esteem when you can't wear … a bathing suit or a white shirt.”

Limitations

The male-female ratio of the cohort of breast cancer survivors is not representative of the true population of breast cancer survivors, where the male-to-female ratio is less than 1:100 [30]. However, datapoints in which the male-female responses were significantly different were identified and addressed with comparisons.

Next, the data-collection process was a potential source of error as the survey was provided in a digital format, meaning undercoverage of the older population is probable, considering the median age of survey participants was 42 years old, as compared to 61, the national median age of cases from 2004 to 2016 [31]. Survey questions, though crafted with the intention of reducing bias, may have also caused response bias from the patients through phrasing and tone. Furthermore, the small sample size (n=100) of the study increases the likelihood of error due to chance.

Furthermore, though analyses were done with various factors in mind, there are several confounding variables that may have influenced the results that were not analyzed. Notable factors include cancer stage and type, which have a large impact on treatments taken and general body condition during and after recovery. Mastectomy type, which greatly affects recovery time and treatment aftereffects, was also not considered.

## Conclusions

Breast cancer and mastectomy have large and lasting impacts on survivors’ lifestyles. They often experience shifts in their goals and values and changes in aspects of their daily lives, including physical and mental health, career, and relationships. Unfortunately, many of the changes they experience are detrimental to their QoL. This study highlights the experiences of breast cancer survivors who underwent mastectomy with the intention of distinguishing in which aspects survivors need the most support so that the community can take effective action towards supporting them, not only medically but in all facets of daily life.
